# Energy choices to health outcomes: A multidimensional analysis of risk in BRICS via PMG-ARDL approach

**DOI:** 10.1371/journal.pone.0310558

**Published:** 2024-12-17

**Authors:** Funda Kaya, Liton Chandra Voumik, Mamunur Rashid, Salma Akter, Sayeem Hasan Khan, Mahdi Salehi, Konrad Kochański, Grzegorz Zimon

**Affiliations:** 1 Department of Environmental Health, Aydin Adnan Menderes University, Efeler, Aydin, Turkiye; 2 Department of Economics, Noakhali Science and Technology University, Noakhali, Bangladesh; 3 Department of Information Technology, School of Business & Technology, Emporia State University, Emporia, KS, United States of America; 4 Department of Economics and Administrative Sciences, Ferdowsi University of Mashhad, Mashhad, Iran; 5 Institute of Economics and Finance, University of Szczecin, Szczecin, Poland; 6 Faculty of Management, Rzeszow University of Technology, Rzeszow, Poland; Islamia University of Bahawalpur, PAKISTAN

## Abstract

This article employs a Panel Mean Group Autoregressive Distributed Lag (PMG-ARDL) approach to investigate the interaction between carbon dioxide (CO_2_) emissions, Gross Domestic Product (GDP), fossil fuel, renewable energy consumption, trade, and their collective impact on life expectancy within the BRICS nations. The research reveals compelling findings. Notably, CO_2_ emissions and trade openness exhibit negative and statistically significant impact on life expectancy. In contrast, GDP per capita and renewable energy consumption are positive and significant determinants of longer life expectancy. The nuanced outcomes underscore the complex interplay of economic, environmental, and social factors within the BRICS nations. The effects found by PMG-ARDL and FMOLS are very comparable, except for the trade openness’ coefficients, which is the inverse. These findings hold significant implications for policy interpretation and sustainable development strategies. As nations struggle to balance economic growth and environmental improvement with public health, tailored interventions targeting CO_2_ reduction, trade openness, renewable energy, and GDP growth can collectively contribute to longer life expectancy. In a broader context, this research contributes to the global discourse on sustainability, economic improvement, and health issue.

## 1. Introduction

In the modern era, many people appeal to enjoy a healthy and better quality of life in suitable environment. Life expectancy, popularly used as a proxy of people’s health, is a crucial indicator in determining living standards, well-being, and human health across the globe. Thus, life expectancy has recently been seriously concerned for climate change issues, environmental degradation, and energy consumption that affect public health and living standards [1–3]. Several studies prove that a nation’s economic, demographical, and ecological conditions influence life expectancy [1, 4–6]. The BRICS countries, consisting of five emerging major economies: China, India, South Africa, Brazil, and Russia, make massive contributions to the world’s energy usage and carbon emissions. According to the statistics compiled by World Bank, in 2021, China’s average life expectancy will be 78.21 years, 72.75 years in Brazil, and 69.36 years in Russia. In contrast, the life span in South Africa and India will be 62.34 and 67.24 years, respectively. As aforementioned, life expectancy is subject to some changes over time for several reasons, such as the improvement of healthcare facilities, changes in lifestyle, advancement in living circumstances, energy utilization, and economic development.

Energy Consumption directly impacts economic and infrastructural development, influencing various aspects of health, well-being, and quality of life. However, greenhouse gases and other pollutants emissions often soar due to after-effects of higher energy usage, which further contribute to air and water pollution, enhance mortality rates, and shorten life expectancy. Energy usage boosted economic activity as well as elevated the levels of CO_2_ emissions [7–10]. The procedure of generating energy, for instance burning, is detrimental to the natural environment since it releases CO_2_, which harms the environment. Global warming and climate change have been investigated primarily due to carbon dioxide emissions and fossil fuel usage by human activity. The Global Energy Statistical Yearbook [11] reported that China emitted the highest carbon emissions worldwide from the BRICS nations, and its total carbon emissions were recorded at 28.8% of the global emissions in 2020, with India’s emissions making up 7.3%, the third highest in the world. Besides, South Africa, Russia, and Brazil each experienced carbon emissions of 4.5%, 1.4%, and 1.3%, correspondingly. The BRICS countries’ combined carbon emissions in 2020 accounted for 43.3% of all carbon emissions worldwide. Traditionally, CO_2_ emissions contribute to air pollution, environmental hazards, and various deleterious effects on the health of individuals [12–14]. What is more, Fossil fuel consumption, especially in the guise of burning coal, natural gas, and oil, significantly negatively influences life expectancy [15–17]. The BRICS are the key energy users of oil, coal, and gas; consequently, fossil fuel energy consumption will remain within 2040 [18]. Regarding the ENERDATA report [19] the total amount of coal burned by the BRICS countries turned out to be 5,217 metric tonnes, the total oil usage was 1,138 metric tonnes, and the total gas consumption was 910 billion cubic meters. So, vast amounts of fossil fuels have improved temperatures and greenhouse gases that induce climate change [20]. Burning pollutants like fossil fuels is a vast source of contamination of air quality and greenhouse gas emissions, leading to various health problems and premature mortality [21]. However, renewable energy consumption is beneficial to mitigate the negative consequences of fossil fuel consumption and essential for better public health and life expectancy. Promoting the usage of renewable energy can aid in mitigating environmental deterioration and extending life expectancy [22]. The transition from non-renewable energy to renewable energy sources such as solar, wind, and hydroelectric power can reduce environmental pollution, leading to cleaner air, fewer diseases, and a longer life span [23].

Subsequently, life expectancy instigates a vital role in achieving stable economic growth [24–27]. In the past few decades, BRICS countries have achieved tremendous economic growth, but unequal income distribution and access to primary resources affect human health. According to the International Monetary Fund’s (IMF) estimation, Brazil’s GDP is projected to be $2.08 trillion in 2023, $3.76 trillion in India, and $19.37 trillion in China. Russia’s GDP was estimated at $2.21 trillion in 2022, and South Africa’s GDP is projected to be $399.01 billion in 2023. The BRICS are predicted to contribute more than 50% of global GDP within 2023, up from 31.5% in 2020 (International Monetary Fund, 2023) [28]. Several studies, for example, Gulis [29]; Kim [30]; Jafrin et al. [31]; Schwandt et al. [32], detected that the earnings per person were indisputably linked to the expectation of life. Children from wealthy families tend to have better health and lower rates of infant and child mortality [33]. This research aims to elucidate the intricate interplay between GDP, renewable and nonrenewable energy consumption patterns, environmental impacts and their collective influence on health outcome within the BRICS countries. By incorporating both economic, energy, and non-economic social factors, this study seeks to provide a comprehensive assessment of the diverse elements shaping life expectancy at birth. This approach offers a more complete view of the contributors to extended lifespan. Beyond correlations, this research endeavors to probe into the causal links between life expectancy and the identified independent variables. This analysis enriches the comprehension of the underlying dynamics and potential pathways for policy intervention.

The contributions of this study to the literature are stated below. (i) First, life expectancy was used as the health outcome in this study. While estimating the factors affecting life expectancy, both economic factors and some non-economic social reasons are included in the econometric model. By employing life expectancy at birth as the focal health indicator, this research merges economic determinants with broader societal influences. This holistic perspective enhances the understanding of longevity determinants. (ii) Unlike previous studies, this research uniquely dissects the effects of renewable and nonrenewable energy consumption on life expectancy within a unified model. This novel approach offers fresh insights into the distinct contributions of these energy types. (iii) The application of both PMG-ARDL and FMOLS methodologies represents a significant contribution to this research. PMG-ARDL offers a robust framework for analyzing the long-term relationships between variables in panel data, allowing for a comprehensive understanding of the intricate dynamics shaping life expectancy in BRICS nations. Meanwhile, FMOLS provides an additional layer of validation, ensuring the reliability and consistency of our findings. This dual-method approach enhances the rigor and credibility of our study, providing valuable insights into the nuanced connections between economic, environmental, and health factors. (iv) In this study, the causality relationship between life expectancy and independent variables was also examined.

This study is designed into five distinct segments: subsequently, the introduction part, the related literature in section 2: “Literature review”, the data and research method in section 3: “Methodology”, the result analyses and explanation in section 4: “Results and Discussion”, and the conclusion with a policy implication drawn in the section 5: “Conclusions” and 6: “Policy Recommendations”. At last the limitations and future research in section 7: “Limitations and Future Research”.

## 2. Literature review

Prior research on the assessment of carbon-dioxide emissions, income, and energy consumption on life expectancy has produced diverse results depending on the employed methodologies, timespans, and countries being considered.

### 2.1. CO_2_ emissions and life expectancy

Numerous research has demonstrated the detrimental effects of carbon emissions on life expectancy. As better health is necessary for emerging countries, it is also crucial to comprehend the factors that affect one’s natural life. Ahmad et al. [34] inspected the connection among socio-economic factors, health, and ambient quality by applying data in China from 1960–2014. According to the results, natural gas, coal, and oil were chosen as environmental quality determiners, and they determined that these contaminants had long-term adverse effects on health. Landrigan et al. [35] explained that the mortality rate was more significant in countries with low or middle incomes than higher-income countries due to larger CO_2_ emissions. It was also suggested that higher pollution negatively affected life expectancy. Asongu [36] examined the impact of increasing carbon dioxide emissions on human health enhancement from 2000–2012 in 44 SSA countries. The investigation proved that CO_2_ emissions were harmful to human health. Hill et al. [37] evaluated whether CO_2_ emission had a ruinous effect on the longevity of life in 49 US states from 2000 to 2010. They found that CO_2_ emissions imposed an unfavourable effect on people’s health. Agbanike et al. [38] applied the ARDL bound test to explore the impact of environmental contaminations and life’s duration from 1971 to 2014 in Nigeria. They identified that CO_2_ emissions had a harmful effect on life expectancy.

Mohammed et al. [39] identified the linkage between economic growth, development, and health in the selected emitting nations. They discovered a strong connection between these factors. They suggested reducing CO_2_ emissions to achieve better life expectancy. Bighlii et al. [40] inspected the association between healthcare expenditure, economic upswing, and environmental degradation in 36 Asian nations. The study discovered through applying panel data that investments in the health care of public and private sectors could lower CO_2_ emissions, which created better environmental quality. Adebayo et al. [41] accessed health, ICT, and CO_2_ connection for the top ten ICT countries from 1986 to 2019. They discovered a detrimental effect of CO_2_ on the environment and life expectancy. Additionally, they showed that most nations indicated a beneficial effect of ICT in reducing CO_2_ emissions. Rahman et al. [42] examined whether environmental deterioration posed a risk to human longevity over time. Their finding also revealed that the higher carbon emission would lead to a lower duration of life in major polluted nations. Mahalik et al. [43] ascertained the linkage between CO_2_ emissions and life expectancy for 68 emerging nations from 1990–2017. They identified how CO_2_ emissions deteriorated life expectancy. Hence, they observed a positive linkage between life expectancy and CO_2_ emissions in 39 emerging countries. Adebayo et al. [44] empirically discovered the relationship between financial globalization and CO_2_ emissions in G7 countries from 1970 to 2018. Most of the findings showed a negative effect of globalization on CO_2_ emissions. They suggested that limiting CO_2_ emissions for better life expectancy was crucial.

Polcyn et al. [45] used the CS-ARDL approach to evaluate the connection between health expenditure, energy consumption, CO_2_ emissions, population size, and income on health in 46 Asian nations from 1997–2019. They found that higher investment in healthcare improved life expectancy. They also showed that CO_2_ emissions were detrimental to health. Das and Debnath [46] employed ARDL bound technique to reinvestigate the connection between CO_2_ emission and life expectancy from 1991–2018 in India. They discovered a strong impact of CO_2_ emission on life expectancy. Consequently, CO_2_ emissions bring down life expectancy.

### 2.2. GDP and life expectancy

Shah et al. [47] results indicate the association between GDP, Life expectancy, and economic growth in G7 Countries from 1960 to 2017. They demonstrated that a higher life expectancy was correlated with a larger GDP per capita income. They also included that a higher life expectancy led to higher spending on health, which affected per capita real income. Liu et al. [48] ascertained China’s economic growth and life expectancy outcomes from 1960 to 2010. The findings revealed that higher economic advancement led to an extension in life expectancy. Asghar et al. [49] identified the linkage between economic expansion and life expectancy by employing the ARDL bounds method in Pakistan from 1972 to 2017. They disclosed a favourable impact of economic prosperity on life expectancy. Murthy et al. [50] discovered how per capita income affected life expectancy between 1992 and 2017 by observing D-8 nations. They outlined that income substantially affected human health as it improved life expectancy. Hendrawati et al. [51] revealed the correlation between income and life span in ASEAN countries from 1988 to 2018. They explored the idea that income ultimately lengthened life expectancy. They also found that a higher living standard could raise life expectancy. Rahman et al. [52] used annual data from 1996 to 2019 to analyse the nexus of people’s earnings with life expectancy in ANZUS-BENELUX countries. They found that income exerts a health-promoting effect. They also emphasized that economic growth improved health care. Azam et al. [53] explained how income influenced people’s longevity from 1975–2020 in Pakistan. The findings of the empirical analysis ultimately concluded that income enhances life expectancy over the long term.

### 2.3. Energy consumption and life expectancy

Recently, related literature has included the effect of non-renewable energy on life expectancy due to extreme fossil fuel usage, which boosts the probability of mortality. Nadimi et al. [54] examined non-renewable energy, which negatively influences people’s health in Japan. This impact depends on the cost of climate change in the future and various strategies for reducing environmental pollution. Hanif [55] investigated the linkage between energy sources and people’s health using the generalized method of moments (GMM) technique in Sub-Saharan Africa. Using fossil fuels harms life expectancy in Sub-Saharan African nations as it increases the death rate. Martins et al. [56] reported the impact of fossil fuel consumption on health in European countries. They found that fossil fuels were the primary economic drivers in many regions, which also denoted a remarkable influence on human health. Koengkan et al. [57] inquired about how renewable energy usage could help diminishes outdoor air pollution and death rates. They discovered a considerable impact of fossil fuels depletion on people’s mortality rate. Ibrahim [58] explored the association between income level, non-renewable fuel sources, life expectancy, and African carbon emissions. They identified fossil fuels as being the leading source of energy consumption. They also found that fossil fuels and carbon emissions decrease life expectancy. In OECD countries, Mujtabe and Sahazad [59] evaluated the association between air quality degradation, economic upturn, and human health. They revealed a causal relationship between renewable energy sources and healthcare spending eventually. Majeed et al. [60] reviewed a study in 155 economies to examine the fastening between renewable energy depletion and health outcomes using GMM in 155 nations. They empirically discovered that renewable energy sources were beneficial to a long-life span. Rodriguez [61] evaluated the nexus of air quality and life expectancy by assessing the need for renewable energy in European countries. They discovered that spending on renewable energy had a beneficial impact on life expectancy. Karimi et al. [62] employed quantile regression to examine the role of renewable energy resources on life expectancy for G-7 countries. They discovered that renewable energy consumption and spending on health improved life expectancy, while carbon dioxide emissions shortened it.

### 2.4. Trade and life expectancy

According to previous research, trade openness can influence life expectancy through many factors. Trade openness generates higher income, influencing economic growth and better life expectancy. Herzer [63] demonstrated the causal connection between trade and public health in 74 countries. They found trade openness’s beneficial impact on life expectancy and infant death rates. Sakyi et al. [64] explored the linkage between the free trade zone and African social welfare. They discovered that trade openness influenced health as tax revenues from trade activities affected the government’s expenditure on health services. Majeed and Qadir [65] evaluated the impact of trade openness on health in Pakistan. They concluded that trade openness had an adverse influence on life expectancy. Novignon et al. [66] investigated the linkage between health and trade openness in sub-Saharan Africa. They explained that trade openness raised life expectancy and decreased infant mortality. Dithmer and Abdulai [67] applied a panel cointegration method to evaluate the connection between trade openness and children’s health. They reviewed that trade lowered the children’s average mortality rate. They also found that a nation with superior institutions and minimal corruption was essential for better health. Shafi & Fatima [68] reported the connection between CO_2_ emissions, trade liberalization, and life expectancy in China. They revealed that trade hampered life expectancy and raised CO_2_ emissions. They implied that renewable energy could offer safe environment and increase life expectancy. Bouchoucha [69] evaluated the linkage between trade openness, CO_2_ emissions, and the lifespan in 49 African nations from 1990–2019. They disclosed a detrimental influence of trade openness on life expectancy. Additionally, the consequences of CO_2_ on health outcomes are highly detrimental. Upon a comprehensive analysis of the existing literature, it is evident that only a limited number of research have examined the potential consequences of energy consumption, economic growth, and human activity on health outcomes, specifically within the BRICS countries. Furthermore, the relationship between economic growth and its impact on health outcomes still needs to be explored. Furthermore, there is a need to investigate the interconnection between CO_2_ emissions, the utilization of renewable energy sources, the promotion of trade liberalization, and the impact on economic growth, all of which influence life expectancy. This study can provide insights into policy initiatives that prioritize human well-being, foster equitable economic development, safeguard the environment, and improve life expectancy. This study addresses the existing research gap by employing the PMG-ARDL approach. No research within the BRICS region has employed this methodology to assess the environmental implications. This section will discuss previous research conducted in the field, and the present work aims to address the gaps identified in these studies.

## 3. Methodology

### 3.1. Data

This study investigates the impact of GDP, trade, environmental pollution, renewable and non-renewable energy consumption on life expectancy in BRICS countries. In the study, annual data for the period 1990–2019 were used, and the World Bank database and Our World in Data were applied to obtain the data. The descriptive information of the variables is given in Table 1.

**Table 1:** Data description and sources.

Variables	Abbreviation	Details and Measurement	Source
Life expectancy	*logLE*_*it*_	Life expectancy, total (year)	WB (2023) [70]
CO_2_ emissions	*logCO*_*2it*_	CO_2_ emissions (per capita metric tons)	WB (2023) [70]
Real income	*logGDP*_*it*_	GDP per capita (constant 2015 US$)	WB (2023) [70]
Fossil fuels energy	*logFOSS*_*it*_	Fossil fuels consumption, per capita (kWh)	Our World in Data (2023) [71]
Renewable energy	*logREC*_*it*_	Renewable energy consumption, per capita (kWh)	Our World in Data (2023) [71]
Trade openness	*logTO*_*it*_	Trade (% of GDP)	WB (2023) [70]

In the study, firstly, descriptive statistics of the series were discussed. The descriptive statistics of the variables are shown in Table 2. As seen in Table 2, the mean value of the *logLE* series is 1.827, 0.559 for the *logCO*_*2*_ series, 3.588 for the *logGDP* series, 1.133 for the *logFOSS* series, 0.051 for the *logREC* series, and 1.575 for the *logTO* series. The variable with the highest maximum value is the *logGDP* series, while the lowest is the *logREC* series. Moreover, the series with the highest minimum value is *logGDP*, while the *logREC* series has the lowest minimum value. Also, the highest standard deviation belongs to the *logREC* series.

**Table 2:** Descriptive statistics.

	*logLE*	*logCO*_*2*_	*logGDP*	*logFOSS*	*logREC*	*logTO*
**Mean**	1.827	0.559	3.588	1.133	0.051	1.575
**Median**	1.832	0.623	3.754	1.152	-0.023	1.624
**Max.**	1.891	1.164	4.006	1.795	0.891	2.043
**Min.**	1.732	-0.188	2.723	0.378	-0.995	1.180
**Std. Dev.**	0.037	0.398	0.375	0.403	0.577	0.174
**Obs.**	150	150	150	150	150	150

Source: Author’s calculation.

**Fig 1:**
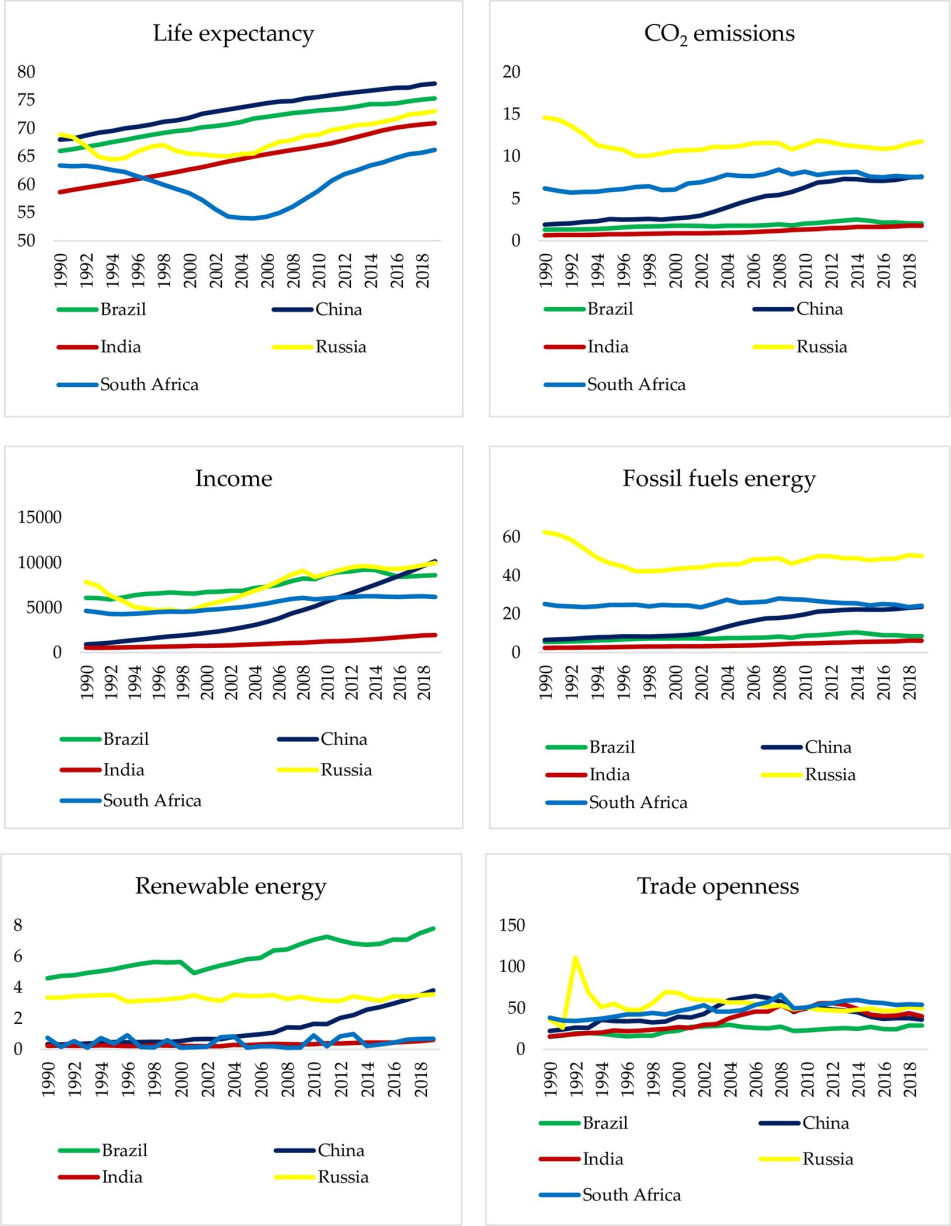
Change of the series used in the study between 1990 and 2019. Sources: World Bank (2023) [70] and Our World in Data (2023) [71].

Graphs of the variables used in the study are presented in Fig 1. According to the graphs in Fig 1, China has the longest life expectancy among the BRICS countries. Russia is the country with the highest CO_2_ emissions among the BRICS countries. As for income per capita, the distribution is more complex and closer to each other. Russia is the country with the highest fossil fuel consumption. Among BRICS countries Brazil consumes the most renewable energy. The trade openness chart is more complex, the data are closer to each other, and the highest fluctuation belongs to Russia.

### 3.2. Theoretical framework

Smith & Dunt [72] postulated a link between various combinations of medical and non-medical inputs and their corresponding output, represented by Eq 1 in his health production function.


HO=f(M.E) (1)

The initials "HO" stands for "health outcomes," "M" for "medical resources," and "E" for "everything" else, including non-medical, social, economic, and lifestyle aspects. It is hypothesized that as healthcare spending enhances (M), so will health outcomes. After a certain point, additional investment in health care is unlikely to yield a significant health improvement. Numerous epidemiological, demographics, and economic research [73, 74], among others, have proposed a wide range of non-medical, social, economic, and physical factors as potential predictors of health conditions.

Therefore, health production depends not just on the health system and its input of resources, but also on non-medical, social, economic, and physical factors. Arawomo et al. [75] employed Smith’s & Dunt’s [72] generalized version of the "health production function" to evaluate the dynamic relationship between economic growth, energy use, and infant mortality in SSA countries. This study has as its theoretical foundation Or’s [76] health production function proposal. Or [76] categorized the non-medical elements into three main groups to facilitate discussion. These include the natural setting, individual choices, and societal dynamics.

This study used life expectancy as a dependent variable, CO_2_ emissions, GDP, fossil fuel, renewable energy consumption, and trade openness as independent variables. The function form of the study is presented below in Eq 2:

$$LE=f\left(CO_{2},GDP,FOSS,REC,TO\right)$$ (2)

In Eq (1), LE denotes the life expectancy (year), CO_2_ emissions represent the carbon dioxide emissions, FOSS denotes the fossil fuel consumption, GDP represents the gross domestic product, REC stands for the renewable energy consumption, TO represents the trade openness.

Therefore, the model implemented during this investigation is defined in terms of the health-generating mechanism. This model has been specified in Eq 3 following the model specification provided by Or [76].


$$LE_{it}=\alpha_{i}+M_{it}\beta +E_{it}\gamma +\mu_{it}$$ (3)

Torras [72] specifies energy consumption and income as the only relevant non-medical elements to consider in this study. Hence, M is a vector of medical variables assessed by trade openness, and E is a vector of non-medical factors typically referred to as ecological variables such as CO_2_. Coefficients on M are denoted by *β* and those on E by γ. It is important to note that γ can be positive or negative, and *β* that can be greater than zero. Health-related subjects of interest are linked in the study. Eq 4 provides a more precise form of health, as mentioned above equation:

$$\begin{array}{l} logLE_{it}=\alpha_{0}+\alpha_{1}logCO_{2it}+\alpha_{2}logGDP_{it}+\\ \alpha_{3}logFOSS_{it}+\alpha_{4}logREC_{it}+\alpha_{5}logTO_{it}+\mu_{it} \end{array}$$ (4)

Here, symbolizes *α*_0_ is the constant term, *α*_1–5_ are coefficients, i represents the cross-section size, t is the time dimension, and μ_*it*_ is the error term.

The variables used in the study were transformed into logarithmic form. When applied to a distribution, the logarithmic adjustment can assist in bringing it closer to the symmetry and normality of a normal distribution. To improve the accuracy of statistical tests, skewed variables might be transformed into normally distributed ones. The logarithmic transformation may additionally be employed to control the variance of a variable, making the analysis more robust against outlying values.

### 3.3. Empirical methods

After the data were organized and defined, the cross-section dependency test was applied to the series’ first in the study. This study uses the Pesaran CSD test for cross-section [77]. Misleading findings, size distortion, consistency bias, and cointegration are just some of the problems that have arisen because of cross-sectional dependence, which needs to be fixed [78–80]. Cross-section dependency testing is crucial when choosing the first-generation or the second-generation unit root tests. The cross-section dependency test should be applied for the results to be dependable and consistent. The following Eq 5 is used for the Pesaran CSD test:

$$CD_{test}=\sqrt{\frac{2T}{N(N-1)}}\left(\sum_{i=1}^{N-1}\sum_{k=i+1}^{N}\hat{\tau_{lk}}\right)$$ (5)

The second-generation unit root tests should be preferred when cross-sectional dependence is detected between series. This study uses the CIPS unit root test for stationarity testing. The CIPS [81] unit root test is explained by the following Eq 6:

$$CIPS\;(N,T)=N^{-1}\sum_{i=1}^{N}t_{i}(N,T)$$ (6)

This study used the PMG-ARDL method to investigate the long-term effects of CO_2_ emissions, real income, fossil fuel and renewable energy consumption, and trade openness on life expectancy. The PMG-ARDL method was proposed by Pesaran et al. [82]. The PMG-ARDL method allows the variables to be stationary at various levels. It is used when the series becomes stationary at I(0), I(1), or a combination of both. The PMG considered a reduced level of heterogeneity, as this enforces homogeneity in the long-term projections and diversity in the short-term estimates. Moreover, it is noteworthy that the PMG can be applied in situations where certain variables exhibit stationarity at the level while others exhibit stationarity at the first difference. Eq 7 defines the estimation of the PMG-ARDL panel paradigm outlined below:

$$\begin{array}{l} \Delta Y_{1it}=\alpha_{1i}+\beta_{1i}Y_{1it-1i}+\sum_{l=2}^{k}\beta_{1i}X_{1it-1}+\sum_{j=1}^{p-1}Y_{1ij}\Delta Y_{1it-j}+\\ \sum_{j=0}^{p-1}\sum_{l=2}^{k}Y_{1ij}\Delta X_{1it-j}+\mu_{it} \end{array}$$ (7)

Here, symbolizes *Y*_1_; dependent variable, Δ*X*_1_; independent variable, *μ*_*it*_; error term, and Δ; the first difference operator.

The paper applied the Dumitrescu Hurlin (D-H) panel causality test for the causality relationship between the series.

To test for causality, D-H causality calculate the individual Wald statistics (*W*_*i*,*t*_) for the cross-section, and then take their arithmetic average and calculate the Wald statistics $\left(W_{N,T}^{HNC}\right)$. D-H causality [83] recommend using test statistics with an asymptotic distribution when T > N and using test statistics with a semi-asymptotic $\left(Z_{N}^{HNC}\right)$ distribution when T < N. The following Eqs 8 and 9 are employed for the D-H causality test:

$$Z_{N,T}^{HNC}=\left(\sqrt{\frac{N}{2K}}W_{N,T}^{HNC}-K\right)$$ (8)

$$Z_{N}^{HNC}=\frac{\sqrt{N}\left[W_{N,T}^{HNC}-N^{-1}\sum_{i=1}^{N}E\left(W_{i,T}\right)\right]}{\sqrt{N^{-1}\sum_{i=1}^{N}\mathrm{var}\left(W_{i,T}\right)}}$$ (9)

## 4. Results and discussion

In the study, a slope heterogeneity test was applied to the series. Table 3 shows the results of the analysis. The p-values show strong evidence against the null hypothesis. In summary, significant slope heterogeneity was detected because of both tests. It is possible to say that the relationship between the variables discussed in the study varies between different groups and subpopulations.

**Table 3:** Slope heterogeneity test results.

Test	Value	p-value
*Δ*	11.840***	0.000
*Δ*_*adj*_	13.522***	0.000

Source: Calculated by the author. *** denotes significance at the 1% level.

This study then applied the Pesaran CSD [81] cross-sectional dependence test to test the cross-sectional dependence between the series. Table 4 shows the Pesaran CSD test results. The p-value of all variables in the Pesaran CSD test results is 0.000. This shows strong evidence against the null hypothesis. The results show that there is a cross-section dependence for all series. It shows that the series in this data set are not independent but that common factors are mutually influenced and influenced by each other.

**Table 4:** CSD test results.

Variables	Pesaran CSD
*logLE*	10.328(0.000) ***
*logCO*_*2*_	7.319(0.000) ***
*logGDP*	15.699(0.000) ***
*logFOSS*	5.832(0.000) ***
*logREC*	6.305(0.000) ***
*logTO*	8.029(0.000) ***

Source: Calculated by the author. *** denotes significance at the 1% level.

After determining the cross-section dependency in all series, we applied the CIPS unit root test, which is one of the second-generation unit root tests to test the stationarity of the series. CIPS unit root test results are presented in Table 5. It is seen that *logLE*, *logCO*_*2*_, *logGDP*, and *logREC* series become stationary after taking the first difference. In addition, it has been determined that the *logFOSS* and *logTO* series are stationary at I (0), that is,level. CIPS unit root test results became stationary after taking all variables’ levels and the first difference in constant and constant + trend. In summary, it is supported by the results that the series has a constant mean and a stable behavior over time.

**Table 5:** CIPS unit root test results.

Variable	Constant		Constant +Trend	
	Level	1^st^ difference	Level	1^st^ difference
CIPS unit root				
*logLE*	-0.877	-3.072***	-2.936**	-3.284***
*logCO*_*2*_	-2.258	-3.435***	-1.393	-3.621***
*logGDP*	-2.179	-2.844***	-1.734	-3.347***
*logFOSS*	-2.404**	-3.632***	-1.754	-3.855***
*logREC*	-1.931	-5.582***	-2.790	-5.769***
*logTO*	-2.699***	-5.078***	-2.651	-5.234***

Source: Author’s calculation. *** and ** refer to significance at 1% and 5% levels.

The PMG-ARDL estimator is the baseline regression to determine the long-term impact of CO_2_ emissions, real income, fossil fuel, renewable energy consumption, and trade openness on life expectancy. The series is stationary at different levels is one reason for choosing the PMG-ARDL method. Table 6 shows the long-term results of PMG-ARDL. In the PMG-ARDL test results, it has been determined that CO_2_ emissions have a negative and statistically significant effect on life expectancy in the long term. Results showed that a 1% increase in CO_2_ emissions will reduce life expectancy by 0.451%. Also, having a relevant effect at the 1% significance level implies solid empirical evidence. Raihan et al. [84]; Nwani et al. [85]; Nkalu and Edeme [86]; Majeed and Ozturk [87]; Nica et al. [88]; Sultana et al. [89]; Alpopi et al. [90]; Nica et al. [91]; Das and Debanth [56]; and Polcyn et al. [45] demonstrate further that life expectancy is reduced in various regions due to CO2 emissions. BRICS countries are among the emerging economies and releasing massive amount of CO_2_. The increase in CO_2_ emissions can cause environmental pollution and deterioration of individual and public health. In addition, the increase in CO_2_ emissions is among the most important causes of air pollution.

**Table 6:** PMG-ARDL long-run result.

Variables	Coefficient	t-Statistic	Prob.
*logCO*_*2it*_	-0.451***	-4.902	0.000
*logGDP*_*it*_	0.639***	21.529	0.000
*logFOSS*_*it*_	0.001	0.011	0.990
*logREC*_*it*_	0.040***	4.397	0.000
*logTO*_*it*_	-0.113**	-2.369	0.019

Source: Author’s calculation. *** and ** show significance at the 1% and 5% levels.

It has been determined that per capita income positively affects life expectancy in the long run and at the 1% significance level. It has been determined that a 1% increase in income increases life expectancy by 0.639%. The increase in the income per capita of individuals in the BRICS countries will affect their lifestyles, spending priorities, and investments in health. At this point, an increase in the income per capita may prolong people’s life expectancy. Murthy et al. [50] investigated the relationship between income and life expectancy in D-8 countries. Through empirical analysis, they found that income increases life expectancy, like our results. Popescu et al [92], Kliestik et al. [93], Ebenstein et al. [94], Messias [95] and Rahman et al. [2018] [96] also demonstrated that rising income accelerated longevity.

The effect of fossil fuel consumption on life expectancy is not statistically significant. In addition, the coefficient is relatively low. It has been determined that fossil fuel consumption does not significantly affect life expectancy in the BRICS countries. Renewable energy has been shown to boost life expectancy in numerous studies, including those by Popescu et al [97], Nica et al [98], Lelieveld et al. [99] and Zimon et al. [100]. The effect of renewable energy consumption on life expectancy is positive and statistically significant. In other words, a positive long-term relationship exists between life expectancy and renewable energy consumption. A 1% increase in renewable energy consumption increases life expectancy by 0.040%. In addition, the significance level of 1% implies strong evidence for the statistical significance of this relationship. When obtaining renewable energy from clean and self-renewing sources and consuming these resources in BRICS countries will contribute to positive results on health. Therefore, this situation shows a curative effect on life expectancy. Renewable energy has been shown to boost life expectancy in numerous studies, including those by Rahman & Alam [101], Popescu et al. [102], Popescu et al. [103], Ghosh et al. [104], Rahman & Alam [42], and Liu & Zhong [26]. The effect of trade openness on long-term life expectancy is negative and statistically significant. It has been determined that there is a negative and long-term relationship between trade openness and life expectancy. A 1% increase in trade openness reduces life expectancy by 0.113%. Fostering trade openness was found to shorten life expectancy, as stated by Bouchoucha [69], Nica [105], Anderi et al. [106], Alam et al. [107], and Govdeli [108]. This finding contradicts Shafi & Fatima [68] which demonstrated that trade expansion accelerated life expectancy.

This study, FMOLS long-term coefficient estimator was used to test the robustness of the PMG-ARDL test results. FMOLS test results are listed in Table 7. We discovered that the *logCO*_*2*_ series had a negative and statistically significant effect on the *logLE* series. We found that the effects of the *logGDP*, *logFOSS* and *logREC* series on life expectancy were like the PMG-ARDL test results. However, we discovered that the *logTO* series had a negative and significant effect at the 1% significance level on the *logLE* series.

**Table 7:** FMOLS test results.

Variables	Coefficient	t-Statistic	Prob.
*logCO*_*2it*_	-0.262***	-6.107	0.000
*logGDP*_*it*_	0.102***	7.567	0.000
*logFOSS*_*it*_	0.194***	4.499	0.000
*logREC*_*it*_	0.023***	4.817	0.000
*logTO*_*it*_	0.048***	5.012	0.000

Note: *** show significance at the 1% levels.

After the PMG-ARDL and FMOLS test results, this study used the D-H panel causality test to examine the causality relationship between. Table 8 gives D-H panel causality test results.

**Table 8:** D-H panel causality test results.

No	Null hypothesis (H_0_)	W- Stat	Zbar- Stat	Prob.	Causality
1	*logCO*_*2*_ *≠logLE*	9.584	11.639	0.000***	*logCO*_*2*_ *→ logLE*
2	*logLE ≠logCO*_*2*_	2.659	2.158	0.030**	*logLE → logCO*_*2*_
3	*logGDP ≠logLE*	9.190	11.099	0.000***	*logGDP → logLE*
4	*logLE ≠logGDP*	8.286	9.862	0.000***	*logLE → logGDP*
5	*logFOSS ≠logLE*	13.391	16.851	0.000***	*logFOSS→ logLE*
6	*logLE ≠logFOSS*	4.761	5.036	0.000***	*logLE → logFOSS*
7	*logREC ≠logLE*	0.769	-0.429	0.667	*None*
8	*logLE ≠logREC*	3.457	3.251	0.001***	*logLE → logREC*
9	*logTO ≠logLE*	3.631	3.488	0.000***	*logTO→ logLE*
10	*logLE ≠logTO*	1.051	-0.043	0.965	None

Source: Author’s calculation. *** and ** show significance at the 1% and 5% levels.

In the D-H panel causality test results, H_0_ was rejected, and a bidirectional causality relationship was detected between CO_2_ emissions and life expectancy. These results show that CO_2_ emissions and life expectancy are caused by each other. Similarly, another bidirectional causal relationship identified between income and life expectancy. The income per capita and life expectancy in BRICS countries is caused by each other. A bidirectional causality has been found between fossil fuel consumption and life expectancy. However, we discovered that there was a unidirectional causal relationship from life expectancy to renewable energy consumption. We determined that there was a unidirectional causality relationship from trade openness to life expectancy.

## 5. Conclusions

Finding the impact of different drivers on life expectancy in the BRICS nations, this study set out to investigate the relationship between CO_2_ emissions, GDP, fossil fuel, renewable energy, and trade openness. Employing the PMG-ARDL and FMOLS approaches, this research unveiled compelling insights into sustainable development and policy formulation within BRICS. CO_2_ emission alleviated life expectancy by 0.451% for 1% enhanced. Trade openness also weakened life expectancy by 0.113%. The adverse impact of CO_2_ and trade openness on life expectancy emphasizes the critical need for policies that balance economic development with environmental conservation. The positive influence of GDP and renewable energy signifies the potential of economic advancement and cleaner energy sources to enhance life expectancy. The GDP and renewable energy enhanced life expectancy by 0.639% and 0.040%, correspondingly, with a 1% boost in GDP and renewable energy. Unexpectedly, the increasing role of fossil fuel consumption shows positive impact on life expectancy, the findings is significant for FMOLS method and insignificant for PMG-ARDL.

D-H panel causality test was used to explore the causality relationship between the dependent and independent variables. Empirical evidence showed a bidirectional causal relationship between CO_2_ emissions, the income per capita and fossil fuel consumption, and life expectancy. In addition, from life expectancy to renewable energy consumption a unidirectional causal relationship has been identified from trade openness to life expectancy.

The results strengthen economic, environmental, and social interdependence in shaping the environment, development, and health interconnection. As governments and policymakers navigate the intricate challenges of sustainable growth, the results of this study offer valuable guidance. In the broader context of global efforts to achieve the United Nations SDGs, this research paper contributes to the ongoing dialogue on sustainable development by providing quantitative results within the BRICS countries. By highlighting the impact of different drivers in shaping life expectancy, this study underscores the necessity of broader policy frameworks that prioritize economic progress and the well-being of citizens.

### 6. Policy recommendations

The results demonstrate that life expectancy in the BRICS is declining due to CO_2_ emissions. The BRICS countries need to take necessary steps in the future to implement policies that reduce pollution and enhance health outcomes. Firstly, they should start implementing and enforcing strict emission control measures across all industry sectors, including setting emissions limits and reduction targets. Such regulatory frameworks should be supplemented by robust monitoring mechanisms and punitive measures for non-compliance, engendering a salient incentive for emission reduction. Secondly, it is necessary to foster a transition towards greener energy involving the augmentation of capacities of alternative energy and the phased reduction of fossil fuel reliance. Promotion of R&D efforts and offering incentives can accelerate this shift. The third way to minimize CO_2_ emissions is through awareness efforts highlighting the health risks of prolonged CO_2_ exposure. Furthermore, these policies can have a multiplicative effect on one another. With the help of interdisciplinary partnerships and global cooperation, they can diminish the detrimental effect of CO_2_ emissions on longevity, thereby manifesting a salutary effect on the public’s health.

On the other hand, GDP had a positive impact on life expectancy and the coefficient is 0.639***. More progress in economic growth and GDP-generating sectors is needed to further increase life expectancy in the future [109]. Firstly, strengthening GDP can strengthen medical and healthcare systems, upgrade medical facilities, and broaden the accessibility of modern healthcare services. Additionally, prioritizing equitable wealth distribution guarantees that the advantages of economic prosperity are accessible to all segments of society, fostering enhancements in living standards and facilitating healthcare access. Furthermore, fostering comprehensive educational initiatives on health, hygiene, and preventive care, supported by an upward trend in GDP, empowers individuals to make well-informed decisions regarding their lifestyle choices conducive to better health. Also, it is imperative to allocate resources toward studies and development endeavours to tackle prevailing health issues and advance groundbreaking medical interventions. Collaborative efforts between governmental bodies, private enterprises, and civil society can synergistically leverage boosted GDP to propel the advancement of public health initiatives. A judicious confluence of these measures, underpinned by a commitment to equitable development, holds promise in capitalizing on the affirmative association between GDP and life expectancy, culminating in enhanced overall welfare and societal advancement.

To capitalize on the empirically documented positive association between renewable energy utilization and life expectancy in BRICS, crafting practical policy recommendations is significant in harnessing this relationship while boosting public health. Firstly, fostering environment conducive to mainstream clean power necessitates implementing targeted incentives and regulatory structures. These mechanisms can encompass tax incentives, subsidies, and streamlined permitting processes to facilitate the integration of energy-efficient technologies. Furthermore, it is crucial to prioritize initiatives to conduct research and development in green power production. Financial resources can facilitate progress in power storage, grid integration, and efficiency improvements, promoting the development of a stronger and more resilient renewable energy landscape. Furthermore, it is imperative to emphasize the significance of public understanding and educational efforts regarding the health advantages associated with renewable energy sources. Citizens with knowledge and awareness can actively support and promote laws about renewable energy, diminishing their dependence on fossil fuels and ameliorating the adverse health consequences associated with such reliance. Collaborative partnerships between governmental entities, private sectors, and research institutions can catalyze the adoption of renewable energy sources, contributing to prolonged life expectancies and improved public health.

Given the mixed relationship between trade and life expectancy, it is advisable to propose policy recommendations to mitigate the potentially unfavourable consequences. To begin with, it is imperative to develop a sophisticated approach toward trade policies that highlights the integration of health considerations in conjunction with economic aims. Striking a balance between economic prosperity and public health necessitates the formulation of trade agreements that incorporate stringent ecological and health standards, thus alleviating the potential negative externalities on life expectancy. Secondly, investing in robust healthcare infrastructure and universal access to quality healthcare services assumes paramount significance. The revenue generated from trade activities could be channelled towards strengthening healthcare systems, remodelling medical facilities, and augmenting health services accessibility, thereby increasing the overall well-being of citizens. Cross-sectoral collaboration between trade and health ministries is instrumental in aligning policy priorities and ensuring that trade openness aligns with health requirements. Eventually, a harmonized and comprehensive approach to trade policies, underpinned by a firm commitment to public health enhancement, holds promise in counteracting the observed detrimental influence of trade openness on life expectancy, thereby fostering more equitable and sustainable socioeconomic development.

## 7. Limitations and future research

Despite the valuable contributions of this research, several limitations need to discuss. Firstly, the study’s focus on the BRICS countries may limit the generalizability of findings to other economies. Secondly, the analysis primarily utilizes aggregate data, potentially overlooking subnational variations that could affect the observed relationships. Additionally, the PMG-ARDL approach assumes homogeneity of coefficients across countries, potentially neglecting unique country-specific dynamics.

Future research will extend and refine this study in various ways. Firstly, exploring individual country analyses within the BRICS could unveil nuanced variations in the identified relationships. Secondly, incorporating diverse data, such as region-specific indicators or sectoral contributions, may improve the accuracy of the analysis. Thirdly, a longitudinal approach could offer insights into the dynamic evolution of the examined variables over time. Moreover, expanding the study to encompass a broader spectrum of emerging economies would provide a more comprehensive understanding of the interplay between economic development, energy utilization, trade, and health outcomes.

## Supporting information

S1 Appendix List of abbreviations. (DOCX)

## References

[1] Blinova T., Bylina S., & tor Rusanovskiy V. (2021). Interregional differences of life expectancy in rural Russia-Assessment of socioeconomic, demographic, behavioral and ecological factors. Geospatial Health, 16(1). doi: 10.4081/gh.2021.876 33706496

[2] Cervellati M., & Sunde U. (2011). Life expectancy and economic growth: the role of the demographic transition. Journal of economic growth, 16, 99–133. doi: 10.1007/s10887-011-9065-2

[3] Pattak D.C.; Tahrim F.; Salehi M.; Voumik L.C.; Akter S.; Ridwan M.; et al. The Driving Factors of Italy’s CO2 Emissions Based on the STIRPAT Model: ARDL, FMOLS, DOLS, and CCR Approaches. Energies 2023, 16, 5845. 10.3390/en16155845

[4] Zimon G.; Tarighi H.; Salehi M.; Sadowski A. Assessment of Financial Security of SMEs Operating in the Renewable Energy Industry during COVID-19 Pandemic. Energies 2022, 15, 9627. 10.3390/en15249627

[5] Hendryx M., & Holland B. (2016). Unintended consequences of the Clean Air Act: Mortality rates in Appalachian coal mining communities. Environmental Science & Policy, 63, 1–6. 10.1016/j.envsci.2016.04.021

[6] Zimon G.; Zimon D. The Impact of Purchasing Group on the Profitability of Companies Operating in the Renewable Energy Sector—The Case of Poland. Energies 2020, 13, 6588. 10.3390/en13246588

[7] Liu T., Yang S., Peng R., & Huang D. (2021). A geographically weighted regression model for health improvement: insights from the extension of life expectancy in China. Applied Sciences, 11(5), 2022. 10.3390/app11052022

[8] Salehi M.; Fahimifard S.H.; Zimon G.; Bujak A.; Sadowski A. The Effect of CO_2_ Gas Emissions on the Market Value, Price and Shares Returns. Energies 2022, 15, 9221. 10.3390/en15239221

[9] Anser M. K., Hanif I., Vo X. V., & Alharthi M. (2020). The long-run and short-run influence of environmental pollution, energy consumption, and economic activities on health quality in emerging countries. Environmental Science and Pollution Research, 27, 32518–32532. doi: 10.1007/s11356-020-09348-1 32506415

[10] Ighodaro C. A. (2010). Cointegration and causality relationship between energy consumption and economic growth: further empirical evidence for Nigeria. Journal of Business Economics and Management, (1), 97–111.

[11] WB (2023). World development indicators database. World Bank, Washington, DC. Retrieved from https://databank.worldbank.org/source/world-development-indicators

[12] Chaabouni S., & Saidi K. (2017). The dynamic links between carbon dioxide (CO2) emissions, health spending, and GDP growth: A case study for 51 countries. Environmental Research, 158, 137–144. doi: 10.1016/j.envres.2017.05.041 28624630

[13] Chaabouni S., Zghidi N., & Mbarek M. B. (2016). On the causal dynamics between CO2 emissions, health expenditures, and economic growth. Sustainable cities and society, 22, 184–191. 10.1016/j.scs.2016.02.001

[14] Mehmood U.; Agyekum E.B.; Kamel S.; Shahinzadeh H.; Moshayedi A.J. Exploring the Roles of Renewable Energy, Education Spending, and CO_2_ Emissions towards Health Spending in South Asian Countries. Sustainability 2022, 14, 3549. 10.3390/su14063549

[15] Ali M., & Seraj M. (2022). Nexus between energy consumption and carbon dioxide emission: evidence from 10 highest fossil fuel and 10 highest renewable energy-using economies. Environmental Science and Pollution Research, 29(58), 87901–87922. doi: 10.1007/s11356-022-21900-9 35821330

[16] Lelieveld J., Klingmüller K., Pozzer A., Burnett R. T., Haines A., & Ramanathan V. (2019). Effects of fossil fuel and total anthropogenic emission removal on public health and climate. Proceedings of the National Academy of Sciences, 116(15), 7192–7197. doi: 10.1073/pnas.1819989116 30910976 PMC6462052

[17] Taghizadeh-Hesary F., & Taghizadeh-Hesary F. (2020). The impacts of air pollution on health and economy in Southeast Asia. Energies, 13(7), 1812. 10.3390/en13071812

[18] BRICS Energy Report. (2021). Retrieved from https://brics2021.gov.in/brics/public/uploads/docpdf/getdocu-41.pdf

[19] Energy Data (2020). https://www.enerdata.net/publications/reports-presentations/world-energy-trends.htm

[20] EPA (2021). EPA Strengthens Key Power Plant Rule to Reduce Smog This Summer and Improve Air Quality for Millions of Americans. Available at: https://www.epa.gov/csapr/revised-cross-state-air-pollution-rule-update.

[21] Trowbridge J., Goin D. E., Abrahamsson D., Sklar R., & Woodruff T. J. (2023). Fossil fuel is the common denominator between climate change and petrochemical exposures and its effects on women’s and childrenʼs health. International Journal of Gynecology & Obstetrics, 160(2), 368–371. 10.1002/ijgo.14408 36069123 PMC9851939

[22] Yılmaz E., & Şensoy F. (2023). Investigating the Causal Relationship between Renewable Energy Consumption and Life Expectancy in Turkey: A Toda-Yamamoto Causality Test. International Econometric Review (IER), 15(1), 1–11. 10.33818/ier.1264805

[23] Caruso G., Colantonio E., & Gattone S. A. (2020). Relationships between renewable energy consumption, social factors, and health: A panel vector autoregression analysis of a cluster of 12 European countries. Sustainability, 12(7), 2915. 10.3390/su12072915

[24] Zhang J., Zhang J., & Lee R. (2001). Mortality declines and long-run economic growth. Journal of Public Economics, 80(3), 485–507.

[25] Alam M. S., Shahbaz M., & Paramati S. R. (2016). The role of financial development and economic misery on life expectancy: Evidence from post financial reforms in India. Social Indicators Research, 128, 481–497. doi: 10.1007/s11205-015-1040-4

[26] Luo W., & Xie Y. (2020). Economic growth, income inequality, and life expectancy in China. Social Science & Medicine, 256, 113046. doi: 10.1016/j.socscimed.2020.113046 32446156

[27] Shahbaz M., Loganathan N., Mujahid N., Ali A., Nawaz A. (2016): Determinants of life expectancy and its prospects under the role of economic misery: a case of Pakistan.–Social Indicators Research 126: 1299–1316 doi: 10.1007/s11205-015-0927-4

[28] International Monetary Fund. (2023). Real GDP Growth. Annual Percent Change. From https://www.imf.org/external/datamapper/NGDP_RPCH@WEO/OEMDC/ADVEC/WEOWORLD/BRA

[29] Gulis G. (2000). Life expectancy as an indicator of environmental health. European Journal of Epidemiology, 16, 161–165. doi: 10.1023/a:1007629306606 10845266

[30] Kim J. I., & Kim G. (2016). Country-level socioeconomic indicators associated with healthy life expectancy: income, urbanization, schooling, and internet users: 2000–2012. Social Indicators Research, 129, 391–402.

[31] Jafrin N., Masud M. M., SEIF A. N. M., Mahi M., & Khanam M. (2021). A panel data estimation of the determinants of life expectancy in selected SAARC countries. Operations Research and Decisions, 31(4), 69–87.

[32] Schwandt H., Currie J., Von Wachter T., Kowarski J., Chapman D., & Woolf S. H. (2022). Changes in the relationship between income and life expectancy before and during the COVID-19 pandemic, California, 2015–2021. JAMA, 328(4), 360–366. doi: 10.1001/jama.2022.10952 35797033 PMC9264223

[33] Frakt A. B. (2018). How the economy affects health. Jama, 319(12), 1187–1188. doi: 10.1001/jama.2018.1739 29584830

[34] Ahmad M., Ur Rahman Z., Hong L., Khan S., Khan Z., and Naeem K. M. (2018). Impact of Environmental Quality Variables and Socioeconomic Factors on Human Health: Empirical Evidence from China. Pollution, 4 (4), 571–579. doi: 10.22059/POLL.2018.252214.391

[35] Landrigan P. J., Fuller R., Hu H., Caravanos J., Cropper M. L., Hanrahan D., et al. (2018). Pollution and global health–an agenda for prevention. Environmental health perspectives, 126(8), 084501. doi: 10.1289/EHP3141 30118434 PMC6108842

[36] Asongu S. A. (2018). CO2 emission thresholds for inclusive human development in sub-Saharan Africa. Environmental Science and Pollution Research, 25, 26005–26019. doi: 10.1007/s11356-018-2626-6 29968214

[37] Hill T. D., Jorgenson A. K., Ore P., Balistreri K. S., & Clark B. (2019). Air quality and life expectancy in the United States: An analysis of the moderating effect of income inequality. SSM-population health, 7, 100346. doi: 10.1016/j.ssmph.2018.100346 30627626 PMC6321951

[38] Agbanike T. F., Nwani C., Uwazie U. I., Uma K. E., Anochiwa L. I., Igberi C., et al. (2019). Oil, environmental pollution, and life expectancy in Nigeria. Applied Ecology Environment Research, 17(5), 11143–11162.

[39] Mohmmed A., Li Z., Arowolo A. O., Su H., Deng X., Najmuddin O., et al. (2019). Driving factors of CO2 emissions and nexus with economic growth, development, and human health in the Top Ten emitting countries. Resources, Conservation and Recycling, 148, 157–169. 10.1016/j.resconrec.2019.03.048

[40] Bilgili F., Kuşkaya S., Khan M., Awan A., & Türker O. (2021). The roles of economic growth and health expenditure on CO2 emissions in selected Asian countries: a quantile regression model approach. Environmental Science and Pollution Research, 28(33), 44949–44972. doi: 10.1007/s11356-021-13639-6 33852118 PMC8045018

[41] Adebayo T. S., Akadiri S. S., Akpan U., & Aladenika B. (2022). The asymmetric effect of financial globalization on carbon emissions in G7 countries: Fresh insight from quantile-on-quantile regression. Energy & Environment, 0958305X221084290. 10.1177/0958305X221084290

[42] Rahman M. M., & Alam K. (2022). Life expectancy in the ANZUS-BENELUX countries: The role of renewable energy, environmental pollution, economic growth, and good governance. Renewable Energy, 190, 251–260. 10.1016/j.renene.2022.03.135

[43] Mahalik M. K., Le T. H., Le H. C., & Mallick H. (2022). How do sources of carbon dioxide emissions affect life expectancy? Insights from 68 developing and emerging economies. World Development Sustainability, 1, 100003. 10.1016/j.wds.2022.100003

[44] Adebayo T. S., Agyekum E. B., Altuntaş M., Khudoyqulov S., Zawbaa H. M., & Kamel S. (2022). Does information and communication technology impede environmental degradation? fresh insights from non-parametric approaches. Heliyon, 8(3). doi: 10.1016/j.heliyon.2022.e09108 35313485 PMC8933682

[45] Polcyn J., Voumik L. C., Ridwan M., Ray S., & Vovk V. (2023). Evaluating the influences of health expenditure, energy consumption, and environmental pollution on life expectancy in Asia. International Journal of Environmental Research and Public Health, 20(5), 4000. doi: 10.3390/ijerph20054000 36901013 PMC10002415

[46] Das S., & Debanth A. (2023). Impact of CO2 emission on life expectancy in India: an autoregressive distributive lag (ARDL) bound test approach. Future Business Journal, 9(1), 5. 10.1186/s43093-022-00179-9

[47] Shah M. I., Ullah I., Xingjian X., Haipeng H., Rehman A., Zeeshan M., et al. (2021). Modeling trade openness and life expectancy in China. Risk Management and Healthcare Policy, 1689–1701. doi: 10.2147/RMHP.S298381 33935523 PMC8079350

[48] Liu H., & Zhong K. (2022). Relationship between health spending, life expectancy, and renewable energy in China: A new evidence from the VECM approach. Frontiers in Public Health, 10, 993546. doi: 10.3389/fpubh.2022.993546 36339134 PMC9631790

[49] Asghar, Wang Z., M. M, Zaidi S. A. H., Nawaz K, Wang B., Zhao W., & Xu F. (2020). The dynamic relationship between economic growth and life expectancy: Contradictory role of energy consumption and financial development in Pakistan. Structural Change and Economic Dynamics, 53, 257–266. 10.1016/j.strueco.2020.03.004.

[50] Murthy U., Shaari M. S., Mariadas P. A., & Abidin N. Z. (2021). The relationships between CO2 emissions, economic growth, and life Expectancy. The Journal of Asian Finance, Economics, and Business, 8(2), 801–808. 10.13106/jafeb.2021

[51] Hendrawaty E., Shaari M.S., Kesumah F.S.D., & Ridzuan A.R. (2022). Economic growth, financial development, energy consumption, and life expectancy: fresh evidence from ASEAN countries. International Journal of Energy Economics and Policy, 12(2), 444–448.

[52] Rahman M.M., Rana R., & Khanam R. (2022). Determinants of life expectancy in most polluted countries: Exploring the effect of environmental degradation. PloS one, 17(1), e0262802. doi: 10.1371/journal.pone.0262802 35061838 PMC8782287

[53] Azam M., Uddin I., & Saqib N. (2023). The determinants of life expectancy and environmental degradation in Pakistan: evidence from ARDL bounds test approach. Environmental Science and Pollution Research, 30(1), 2233–2246. doi: 10.1007/s11356-022-22338-9 35930156

[54] Nadimi R., & Tokimatsu K. (2017). Analyzing renewable and non-renewable energy consumption via Bayesian inference. Energy Procedia, 142, 2773–2778. 10.1016/j.egypro.2017.12.224

[55] Hanif I. (2018). Energy consumption habits and human health nexus in Sub-Saharan Africa. Environmental Science and Pollution Research, 25(22), 21701–21712. doi: 10.1007/s11356-018-2336-0 29790046

[56] Martins F., Felgueiras C., & Smitková M. (2018). Fossil fuel energy consumption in European countries. Energy Procedia, 153, 107–111. 10.1016/j.egypro.2018.10.050

[57] Koengkan M., Fuinhas J. A., & Silva N. (2021). Exploring the capacity of renewable energy consumption to reduce outdoor air pollution death rate in Latin America and the Caribbean region. Environmental Science and Pollution Research, 28, 1656–1674. doi: 10.1007/s11356-020-10503-x 32845465

[58] Ibrahim R. L. (2022). Beyond COP26: Can income level moderate fossil fuels, carbon emissions, and human capital for healthy life expectancy in Africa? Environmental Science and Pollution Research, 29(58), 87568–87582. doi: 10.1007/s11356-022-21872-w 35819679

[59] Mujtaba G.; Shahzad S.J.H. (2020). Air pollutants, economic growth, and public health: Implications for sustainable development in OECD countries. Environ. Sci. Pollut. Res., 28, 12686–12698. doi: 10.1007/s11356-020-11212-1 33085009 PMC7576550

[60] Majeed M. T., Luni T., & Zaka G. (2021). Renewable energy consumption and health outcomes: Evidence from global panel data analysis. Pakistan Journal of Commerce and Social Sciences (PJCSS), 15(1), 58–93.

[61] Rodriguez-Alvarez A. (2021). Air pollution and life expectancy in Europe: Does investment in renewable energy matter? Science of the Total Environment, 792, 148480. doi: 10.1016/j.scitotenv.2021.148480 34153769

[62] Karimi Alavijeh N., Ahmadi Shadmehri M. T., Dehdar F., Zangoei S., & Nazeer N. (2023). The role of renewable energy on life expectancy: evidence from the method of moments quantile regression based on G-7 countries data. International Journal of Energy Sector Management. 10.1108/IJESM-11-2022-0001

[63] Herzer D. (2017). The Long‐run relationship between trade and population health: Evidence from five decades. The World Economy, 40(2), 462–487. 10.1111/twec.12419

[64] Sakyi D., Bonuedi I., & Opoku E. E. O. (2018). Trade facilitation and social welfare in Africa. Journal of African Trade, 5(1–2), 35–53. 10.1016/j.joat.2018.08.001

[65] Majeed M. T. & Qadir N. (2018). The impact of trade liberalization on health: Evidence from Pakistan. Emp. Economy. Rev., 1(1), 71–108.

[66] Novignon J., Atakorah Y. B., & Djossou G. N. (2018). How does the health sector benefit from trade openness? Evidence from Sub‐Saharan Africa. African Development Review, 30(2), 135–148. 10.1111/1467-8268.12319

[67] Dithmer J., & Abdulai A. (2020). Trade openness and child health: A heterogeneous panel cointegration analysis. Applied Economics, 52(23), 2508–2525. 10.1080/00036846.2019.1693018

[68] Shafi R., & Fatima S. (2019). Relationship between GDP, Life expectancy, and growth rate of G7 Countries. International Journal of Sciences, 8(06), 74–79.

[69] Bouchoucha N. (2023). Does Trade Openness and Environmental Quality Matter for Health Status? Evidence from African Countries. Journal of the Knowledge Economy, 1–17.

[70] World Bank. (2023). World Development Indicators. Retrieved February 15, 2023, from http://databank.worldbank.org/source/world-development-indicators

[71] Our World in Data. (2023). Retrieved March 05, 2023, from https://ourworldindata.org/source/the-source-of-the-data

[72] Smith C. S., & Dunt D. R. (1992). The work program of the National Centre for Health Program Evaluation. Australian Health Review, 15(2), 117–123. 10119043

[73] Barro, R. J. (1996). Three models of health and economic growth. Cambridge, MA: Harvard University. Unpublished manuscript.

[74] Torres M. (2005). “Income and Power Inequality as Determinants of Environmental and Health Outcomes”. 86

[75] Arawomo O., Oyebamiji Y. D., & Adegboye A. A. (2018). Dynamics of economic growth, energy consumption and health outcomes in selected sub-Sahara African countries. African Journal of Economic Review, 6(2), 92–114.

[76] Or Z. (2000). Determinants of health outcomes in industrialized countries: a pooled, cross-country, time-series analysis. OECD Economic Studies, 53–78.

[77] Pesaran M. H. (2004). General diagnostic tests for cross-section dependence in panels. Available at SSRN 572504.

[78] Khan D., Nouman M., Popp J., Khan M. A., Ur Rehman F., & Oláh J. (2021). The link between technically derived energy efficiency and ecological footprint: Empirical evidence from the ASEAN region. Energies, 14(13), 3923. 10.3390/en14133923

[79] Khan D., & Ullah A. (2020). Comparative analysis of the technical and environmental efficiency of the agricultural sector: The case of Southeast Asia countries. Custos E Agronegocio Line, 16, 2–28.

[80] Westerlund J., & Edgerton D. L. (2007). A panel bootstrap cointegration test. Economics Letters, 97(3), 185–190. 10.1016/j.econlet.2007.03.003

[81] Pesaran M.H. (2007). A simple panel unit root test in the presence of cross‐section dependence. Journal of applied econometrics, 22(2), 265–312. 10.1002/jae.951

[82] Pesaran M.H., Shin Y., & Smith R. P. (1999). Pooled mean group estimation of dynamic heterogeneous panels. Journal of the American Statistical Association, 94(446), 621–634.

[83] Dumitrescu E.I., & Hurlin C. (2012). Testing for Granger non-causality in heterogeneous panels. Economic modeling, 29(4), 1450–1460.

[84] Raihan A., Rashid M., Voumik L. C., Akter S., & Esquivias M. A. (2023). The Dynamic Impacts of Economic Growth, Financial Globalization, Fossil Fuel, Renewable Energy, and Urbanization on Load Capacity Factor in Mexico. Sustainability, 15(18), 13462.

[85] Nwani S. E., Kelani F. A., Ozegbe A. E., & Babatunde O. H. (2018). Public health expenditures, environmental pollution, and health outcomes: Evidence from Nigeria. South Asian Journal of Social Studies and Economics,2(2) 1–15

[86] Nkalu C. N., & Edeme R. K. (2019). Environmental hazards and life expectancy in Africa: evidence from GARCH model. Sage Open, 9(1), 2158244019830500. doi: 10.1177/2158244019830500

[87] Majeed M. T., & Ozturk I. (2020). Environmental degradation and population health outcomes: a global panel data analysis. Environmental Science and Pollution Research, 27(13), 15901–15911. doi: 10.1007/s11356-020-08167-8 32100215

[88] Nica E., Poliak M., Alpopi C., Kliestik T., Manole C., & Burlacu S. (2023). Impact of Trade, FDI, and Urbanization on Female Employment System in SAARC: GMM and Quantile Regression Approach. Systems, 11(3), 137. 10.3390/systems11030137

[89] Sultana T., Hossain M. S., Voumik L. C., & Raihan A. (2023). Democracy, green energy, trade, and environmental progress in South Asia: Advanced quantile regression perspective. Heliyon, 9(10). doi: 10.1016/j.heliyon.2023.e20488 37822611 PMC10562800

[90] Alpopi C., Nica E., Oancea M. D. N., & Balu P. E. (2019). Aspects Of Sustainable Development in The Perspective of The Population Aging Phenomenon. Quality-Access to Success, 20.

[91] Nica E., & Potcovaru A. M. (2019). The Impact of Motivation on the Work Performance in Health System. In Proceedings of the international management conference (Vol. 13, No. 1, pp. 913–918).

[92] Popescu G. H., Andrei J. V., Nica E., Mieilă M., & Panait M. (2019). Analysis on the impact of investments, energy use and domestic material consumption in changing the Romanian economic paradigm. Technological and Economic Development of Economy, 25(1), 59–81.

[93] Kliestik T., Valaskova K., Nica E., Kovacova M., & Lazaroiu G. (2020). Advanced methods of earnings management: Monotonic trends and change-points under spotlight in the Visegrad countries. Oeconomia Copernicana, 11(2), 371–400.

[94] Ebenstein A., Fan M., Greenstone M., He G., Yin P., & Zhou M. (2015). Growth, pollution, and life expectancy: China from 1991–2012. American Economic Review, 105(5), 226–231.

[95] Messias E. (2003). Income inequality, illiteracy rate, and life expectancy in Brazil. American Journal of Public Health, 93(8), 1294–1296. doi: 10.2105/ajph.93.8.1294 12893617 PMC1447959

[96] Rahman M. M., Khanam R., & Rahman M. (2018). Health care expenditure and health outcome nexus: new evidence from the SAARC-ASEAN region. Globalization and health, 14(1), 1–11. 30466452 10.1186/s12992-018-0430-1 PMC6249744

[97] Popescu G. H., Mieila M., Nica E., & Andrei J. V. (2018). The emergence of the effects and determinants of the energy paradigm changes on European Union economy. Renewable and Sustainable Energy Reviews, 81, 768–774.

[98] Nica E., Sima V., Gheorghe I., & Drugau-Constantin A. (2018). Analysis of Regional Disparities in Romania from an Entrepreneurial Perspective. Sustainability, 10(10), 3450.

[99] Lelieveld J., Klingmüller K., Pozzer A., Burnett R. T., Haines A., & Ramanathan V. (2019). Effects of fossil fuel and total anthropogenic emission removal on public health and climate. Proceedings of the National Academy of Sciences, 116(15), 7192–7197. doi: 10.1073/pnas.1819989116 30910976 PMC6462052

[100] Zimon G., Pattak D.C., Voumik L.C., Akter S., Kaya F., Walasek R., et al. The Impact of Fossil Fuels, Renewable Energy, and Nuclear Energy on South Korea’s Environment Based on the STIRPAT Model: ARDL, FMOLS, and CCR Approaches. Energies. 2023; 16(17):6198. 10.3390/en16176198

[101] Rahman M. M., & Alam K. (2022). Life expectancy in the ANZUS-BENELUX countries: The role of renewable energy, environmental pollution, economic growth and good governance. Renewable Energy, 190, 251–260.

[102] Popescu G. H., Sima V., Nica E., & Gheorghe I. G. (2017). Measuring sustainable competitiveness in contemporary economies—Insights from European economy. Sustainability, 9(7), 1230.

[103] Popescu G. H., & Nica E. (2015). Global warming, climate policy, and the green paradox. In Green economic structures in modern business and society (pp. 20–38). IGI Global.

[104] Ghosh S., Hossain M. S., Voumik L. C., Raihan A., Ridzuan A. R., & Esquivias M. A. (2023). Unveiling the Spillover Effects of Democracy and Renewable Energy Consumption on the Environmental Quality of BRICS Countries: A New Insight from Different Quantile Regression Approaches. Renewable Energy Focus. 10.1016/j.ref.2023.06.004

[105] Nica E. (2020). Buying organic food as sustainable consumer decision-making behavior: Cognitive and affective attitudes as drivers of purchase intentions toward environmentally friendly products. In SHS Web of Conferences (Vol. 74, p. 04018). EDP Sciences.

[106] Andrei J. V., Popescu G. H., Nica E., & Chivu L. (2020). The impact of agricultural performance on foreign trade concentration and competitiveness: empirical evidence from Romanian agriculture. Journal of Business Economics and Management, 21(2), 317–343.

[107] Alam M. S., Raza S. A., Shahbaz M., & Abbas Q. (2016). Accounting for contribution of trade openness and foreign direct investment in life expectancy: The long-run and short-run analysis in Pakistan. Social Indicators Research, 129, 1155–1170. doi: 10.1007/s11205-015-1154-8 32214606 PMC7088970

[108] Gövdeli T. (2019). Life expectancy, direct foreign investments, trade openness and economic growth in E7 countries: Heterogeneous panel analysis. Third Sector Social Economic Review, 54(2), 731–743.

[109] Akter S, Voumik L. C H Rahman M., Raihan A & Zimon G. (2023) GDP, health expenditure, industrialization, education and environmental sustainability impact on child mortality: Evidence from G-7 countries, Sustainable Environment, 9:1, doi: 10.1080/27658511.2023.2269746

