# Supplementary material for: Energy choices to health outcomes: A multidimensional analysis of risk in BRICS via PMG-ARDL approach
Source: PLoS One. 2024 Dec 17;19(12):e0310558. doi: 10.1371/journal.pone.0310558 (PMC11651603; doi:10.1371/journal.pone.0310558)
Supplement: S1 Appendix — (DOCX) [file pone.0310558.s001.docx]

**S1 Appendix:** List of Abbreviations

| Abbreviation | Details |
| --- | --- |
| ARDL | Autoregressive distributed lag |
| BRICS | An acronym for Brazil, Russia, India, China, and South Africa. |
| CO_2_ | Carbon-dioxide emission |
| CD | Cross-sectional dependence |
| CIPS | Cross-section Im-Pesaran-Shin |
| D-8 | An organization comprising Bangladesh, Egypt, Indonesia, Iran, Malaysia, Nigeria, Pakistan, and Turkey to cooperate on development |
| EPA | Environmental Protection Agency |
| FOS | Fossil fuel |
| GDP | Gross Domestic product |
| G7 | Group of Seven, including Canada, France, Germany, Italy, Japan, the United Kingdom, and the United States, as well as the European Union. |
| GMM | Generalized Method of Moments |
| HO | Health outcome |
| IMF | International Monetary Fund |
| ICT | Information, Communication, & Technology |
| LE | Life expectancy |
| logCO_2_ | Log of carbon-dioxide emission |
| logLE | Log of Life Expectancy |
| logGDP | Log of gross domestic product |
| logREC | Log of renewable energy consumption |
| logFOS | Log of fossil fuel |
| logTO | Log of trade openness |
| OECD | Organisation for Economic Co-operation and  Development |
| PMG-ARDL | Panel Mean Group Autoregressive Distributed Lag |
| REC | Renewable Energy Consumption |
| R&D | Research & Development |
| TO | Trade openness |
| WB | World Bank |
