# Supplementary material for: Energy choices to health outcomes: A multidimensional analysis of risk in BRICS via PMG-ARDL approach
Source: PLoS One. 2024 Dec 17;19(12):e0310558. doi: 10.1371/journal.pone.0310558 (PMC11651603; doi:10.1371/journal.pone.0310558)
Supplement: Attachment — Submitted filename: Reviewers Response.docx [file pone.0310558.s002.docx]

**Reviewers Response**

**Reviewer #2:** The following revisions are suggested.
1. It is suggested to revise the title. Otherwise, justify the use of these terms: Energy Choice, Multidimensional Analysis, Risk, with the contents of the study.

**Response:** Thanks Professor, we revised the title.

2. Add one or two sentences, at the very beginning of the abstract, narrating the significance of the study.

**Response:** Thanks, professor. We have provided 2 lines at the beginning of the abstract, that narrate the significance of the study.

3. Several studies have already evidenced health outcomes as a function of economic growth, energy consumption, and carbon emissions. At the same time, many studies have revealed that economic-environmental-social interdependence in shaping the development trajectory. What is the
the novelty of the current study?

**Response:** Thanks, Professor. We have provided the novelty of this paper and marked its by purple color.

4. Economic growth-health outcome is quite logical and empirically testified as well. Energy consumption and carbon dioxide emissions are directly linked to economic growth (through the energy-growth-emission model), hence, indirectly with the health outcome. It seems that the current study has fitted the energy-growth-emission model for the health outcome. It is logical that energy consumption (REC and NREC) in developing economies leads to economic growth, and due to more fossil fuel use (for economic growth) carbon dioxide emission is obvious. So, could there be a way to separate the direct and indirect effects of Energy consumption and carbon dioxide emission on the health?

**Response: Information about the relationship between energy consumption and CO2 emissions was written in the content of the manuscript.**

5. What is the purpose of presenting the Dumitrescu Hurlin panel causality, especially, where the causality moves from life expectancy towards carbon dioxide emissions and fossil fuel consumption.

**Response:** Thanks, Professor. We have provided the purpose of presenting the Dumitrescu Hurlin panel causality, especially, where the causality moves from life expectancy towards carbon dioxide emissions and fossil fuel consumption and is marked by purple color.

6. The study has used the PMG-ARDL and the FMOLS tests. The FMOLS test was used to test the robustness of the PMG-ARDL test results. What is the implication of varying signs of the coefficient logTO in both tests?

**Response: Thanks, Professor. We have provided the implication of varying sign of the coefficient logTO in PMG-ARDL and FMOLS.**

7. The place for the Figure 1 is mentioned but the figure is nowhere in
the manuscript.

**Response: Thanks, Professor. We have added the figure.**

8. A citation "Rahman, M. M., & Alam, K. (2022)" is numbered differently, i.e., as 42 and then as 101 (verify in the complete references).

**Response: Thanks, Professor. We have edited the references.**

-------------------------
